# HIV-1 Tat interacts with LIS1 protein

**DOI:** 10.1186/1742-4690-2-6

**Published:** 2005-02-07

**Authors:** Nicolas Epie, Tatyana Ammosova, Tamar Sapir, Yaroslav Voloshin, William S Lane, Willie Turner, Orly Reiner, Sergei Nekhai

**Affiliations:** 1 Center for Sickle Cell Disease, Howard University, Washington DC 20059, USA; 2 Department of Microbiology, Howard University College of Medicine, 520 W Street N.W., Washington, DC 20059, USA; 3 Department of Biochemistry and Molecular Biology, Howard University College of Medicine, 520 W Street N.W., Washington, DC 20059, USA; 4 Department of Molecular Genetics, The Weizmann Institute of Science, 76100, Rehoboth, Israel; 5 Harvard Microchemistry Facility, 16 Divinity Ave., Cambridge MA 02138, USA

## Abstract

**Background:**

HIV-1 Tat activates transcription of HIV-1 viral genes by inducing phosphorylation of the C-terminal domain (CTD) of RNA polymerase II (RNAPII). Tat can also disturb cellular metabolism by inhibiting proliferation of antigen-specific T lymphocytes and by inducing cellular apoptosis. Tat-induced apoptosis of T-cells is attributed, in part, to the distortion of microtubules polymerization. LIS1 is a microtubule-associated protein that facilitates microtubule polymerization.

**Results:**

We identified here LIS1 as a Tat-interacting protein during extensive biochemical fractionation of T-cell extracts. We found several proteins to co-purify with a Tat-associated RNAPII CTD kinase activity including LIS1, CDK7, cyclin H, and MAT1. Tat interacted with LIS1 but not with CDK7, cyclin H or MAT1 *in vitro*. LIS1 also co-immunoprecipitated with Tat expressed in HeLa cells. Further, LIS1 interacted with Tat in a yeast two-hybrid system.

**Conclusion:**

Our results indicate that Tat interacts with LIS1 *in vitro *and *in vivo *and that this interaction might contribute to the effect of Tat on microtubule formation.

## Background

HIV-1 Tat protein is the viral transactivator encoded in the HIV-1 genome of infected cells [1-3]. Tat stimulates formation of full-length transcripts from the HIV-1 promoter by promoting efficient transcript elongation (reviewed in [4]). Tat interacts with the bulge of transactivation response (TAR) RNA, a hairpin-loop structure at the 5'-end of all nascent viral transcripts [5-7]. Tat induces elongation of HIV-1 transcription by recruiting transcriptional co-activators that include Postive Transcription Elongation Factor b (P-TEFb), an RNA polymerase II C-terminal domain kinase [8-10] and histone acetyl transferases [11-13]. Whereas P-TEFb induces HIV-1 transcription from non-integrated HIV-1 template [8-10], histone acetyl transferases allow induction of integrated HIV-1 provirus [11-13]. Tat may also increase initiation of HIV-1 transcription by enhancing phosphorylation of SP1, a transcription factor involved in the basal HIV-1 transcription [14]. In addition to its function in HIV-1 transcription, Tat may contribute to HIV-1 pathogenesis by regulating signal transduction in endothelial cells [15,16]; functioning as a secreted growth factor for Kaposi sarcoma and endothelial cells [17]; and inducing apoptosis in T-cells by binding to microtubules and delaying tubulin depolymerization [18,19]. Tat induces apoptosis through BIM, a pro-apoptotic protein of the Bcl-2 family that antagonizes Bcl-2 anti-apoptotic proteins [18]. The effect of Tat is similar to the effect of Taxol, a drug that stabilizes microtubules and induces apoptosis [18]. Mutations in the glutamine-rich region of Tat protein (residues 60–72) were found to correlate with rapid progression of HIV disease, and with induction of apoptosis and binding to tubulin [20]. We previously showed that microtubules polymerization is facilitated by LIS1 protein [21], a causative factor for Lissencephaly [22], a severe brain disorder resulted from inefficient neuronal migration during early stages of brain development [23]. LIS1, a 45 kDa protein, contains seven repeating units called WD (Trp-Asp) repeats [24] that form antiparallel sheets making up a toroidal propeller structure [25]. WD repeats containing proteins are confined to eukaryotes and participate in protein-proteins interactions [24]. In addition to being a microtubule binding protein, LIS1 is also a subunit of platelet-activating factor acetyl hydrolase (PAF-AH) [26]. LIS1 interacts with dynein motor, NudC and Dynactin, a complex that regulates microtubule dynamics [27,28]. LIS1 in addition associates with Nudel [29], also a component of the dynein motor complex, and this interaction affects dephosphorylation of microtubules by protein phosphatase 2A (PP2A) [30]. Thus, LIS1 may function as a scaffold that help to assemble dynein motor and serve to regulate proper microtubule dynamics.

In the present paper, we fractionated extracts of Jurkat T-cells using HIV-1 Tat as an affinity bait and RNAPII CTD activity of the Tat-associated proteins as a selection criteria. We identified by mass-spectrometry and immunoblotting components of the partially purified protein fraction and found LIS1, CDK7, cyclin H, and MAT1. We analyzed interaction of Tat with the identified individual proteins and found that Tat interacts with LIS1. We confirmed this finding by co-immunoprecipitating Tat and LIS1 from HeLa cells that were expressing Tat. And we also confirmed binding of Tat to LIS1 in a yeast two-hybrid system. Our results indicate that HIV-1 Tat interacts directly with LIS1, and therefore this interaction might contribute to the effect of Tat on microtubules formation in the cells.

## Results

### LIS1, CDK7, cyclin H, and MAT1 co-purify with Tat-associated RNAPII CTD kinase activity

**Figure 1:**
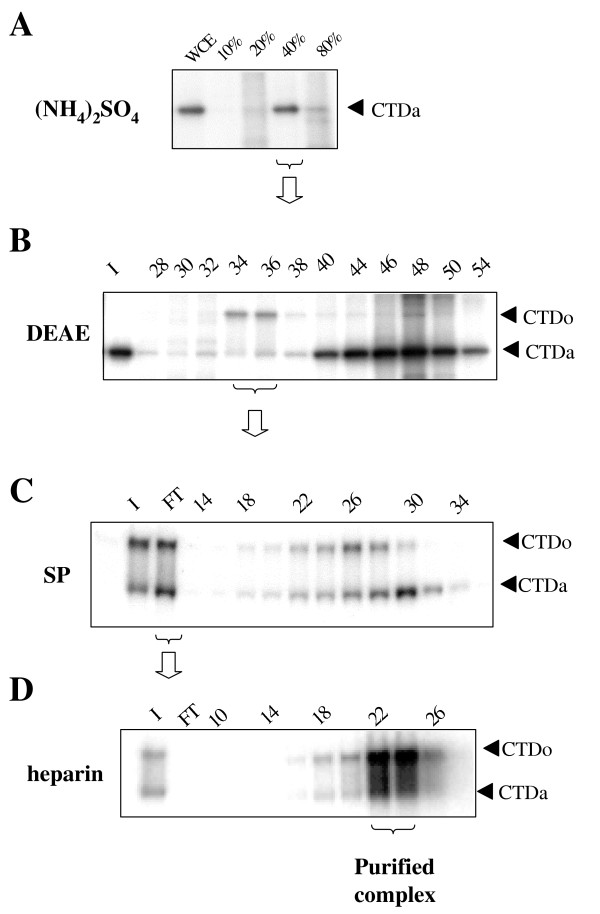
**Purification of Tat Associated CTD Kinase. *A*, Ammonium sulfate fraction of T-cell extract. **Whole cell extract of Jurkat T cells was fractionated by ammonium sulfate added sequentially to 10%, 20%, 40% and 80% saturation as described in *Experimental procedures*. Fractions were analyzed for Tat-associated CTD kinase activity as described in the *Experimental procedures *section. A portion of each fraction was bound to GST-Tat 72 immobilized on glutathione-agarose beads and then incubated with [γ-^32^P] ATP and recombinant GST-CTD. Phosphorylated GST-CTD was resolved on SDS/10%-(w/v)-PAGE. *B*, DEAE-Sepharose column-chromatographic elution profile. Jurkat T-cell extract 40%-(NH_4_)_2_SO_4 _cut was applied to a DEAE-Sepharose column. Fractions were analyzed for Tat-associated CTD kinase activity as described above. *C*, SP-Sepharose column-chromatographic elution profile. DEAE-fractions 32 to 36 containing hyperphosphorylating CTD kinase activity were combined and applied to SP-Sepharose column. *D*, heparin-agarose column-chromatographic elution profile. SP-Sepharose flow-through fraction was collected and further fractionated on Hi Trap heparin column. Fractions 22 to 24 (labelled as purified complex) contained Tat-associated CTD hyperphosphorylating activity. Positions of CTDa and CTDo are shown. The figure is an autoradiogram.

**Figure 2:**
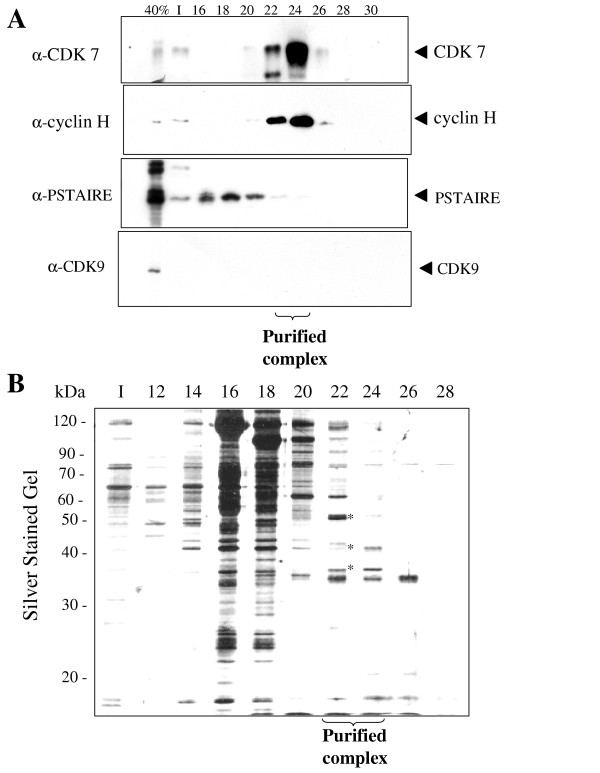
**Analysis of protein composition of heparin-agarose purified fraction of Tat-associated CTD kinase. *A*, Heparin-agarose-purified fraction contains CDK7 but not CDK9. **Fractions from the heparin-agarose column fractionation shown in Fig. 1 were analyzed by Western blotting with antibodies against CDK7, Cyclin H, PSTAIRE and CDK9. Fractions 22 to 24 which contain Tat-associated CTD hyperphosphorylating activity also contain CDK7 and cyclin H, but not CDK9 or PSTAIRE-like kinase. *B*, **Tat-associated CTD kinase co-purifies with 35, 40 and 50 kDa protein bands. **Fractions from the heparin-agarose column fractionation were resolved on 12% SDS PAGE and stained with silver. Protein bands of 35, 40, and 50 kDa that co-purify with the CTD kinase activity are marked by stars.

**Figure 3:**
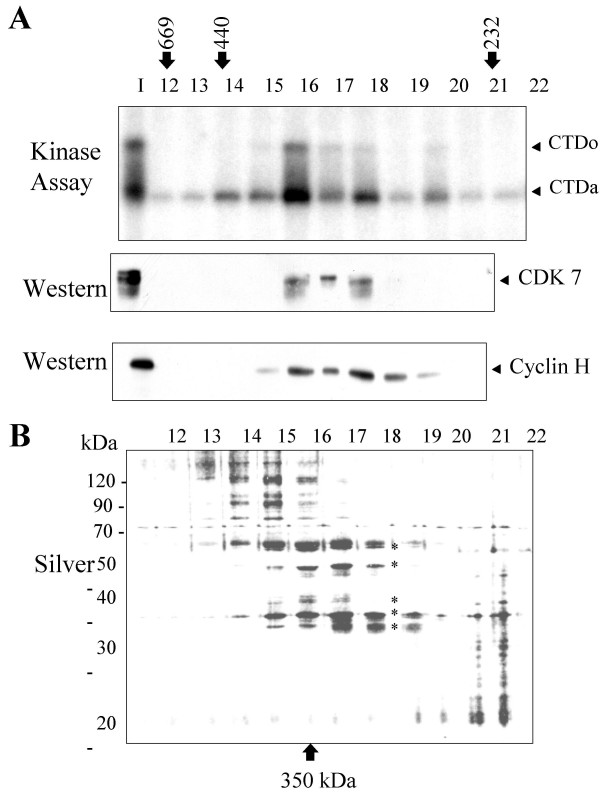
**CDK7 and cyclin H co-migrate as a 350 kDa complex. **Hi-Trap heparin fractions 22 to 24 were analyzed on Sephacryl S-300 gel filtration column. *A*, **Tat-associated CTD kinase activity co-purify with CDK7 and cyclin H. **Fractions from the Sephacryl S-300 column fractionation were analyzed for Tat-associated CTD kinase activity and also by Western blotting with antibodies against CDK7 and Cyclin H. *B*, Fractions from Sephacryl S-300 column fractionation were resolved by 12% SDS PAGE and stained with silver.

We reported previously that HIV-1 Tat associates with two distinct protein kinase complexes purified from mitogenically stimulated human primary T-lymphocytes; one complex containing CDK2 and the other one CDK7 [31]. The CDK2-containing protein complex was previously purified and characterized by us [32,33] and we showed that CDK2 regulates HIV-1 transcription [34]. In the present paper, we purify and characterize the CDK7-containing protein elution peak. Whole-cell lysate from Jurkat T cells was prepared and subjected to (NH_4_)_2_SO_4 _fractionation as described previously [32]. In accord with our previous report [32], the 40% (NH_4_)_2_SO_4 _cut contained Tat-associated CTD kinase activity (Fig. 1A). The 40% (NH_4_)_2_SO_4 _cut was subsequently fractionated on DEAE-Sepharose (Fig. 1B). As we previously reported, separation of the ammonium sulphate cut on DEAE-Sepharose resulted in the appearance of Tat-associated CTD hyperphosphorylating activity (Fig. 1B, fractions 34 to 36). Hyperphosphorylated CTD (CTDo) migrated on SDS-PAGE with a high degree of retardation, because of SDS repelling effect. Immunoblotting of the DEAE-Sepharose fractions 34 to 36 showed the presence of CDK7, CDK9 and a PSTAIRE-motif containing kinase, but not TFIIH (See Additional File 1) in accordance with our previous observations [32]. Further resolution of DEAE fractions 34–36 on SP-Sepharose column showed that part of the Tat-associated CTD kinase activity was retained by the column and we previously identified this activity as containing CDK2 [32]. The other part of the Tat-associated CTD kinase activity was not retained by the column and was eluted as a flow-through fraction (Fig. 1C, flow through fraction). This fraction was collected and further resolved on Hi-Trap heparin column (Fig. 1D). Immunoblotting of the Hi-Trap heparin fractions showed that Tat-associated kinase activity co-eluted with CDK7 and also with cyclin H, but not with CDK9 or a PSTAIRE-motif containing kinase (Fig. 2A). Silver staining of the Hi-Trap heparin fractions showed that fractions 22 and 24 contained three protein bands of 35, 40, and 50 kDa which co-eluted with the Tat-associated CTD kinase activity (Figure 2B; fractions 22 and 24, protein bands marked by stars). The Hi-Trap heparin fractions 22 to 24 were further analyzed on Sephacryl S-300 gel filtration column to determine whether CDK7, cyclin H and unknown protein bands comigrate as a single macromolecular mass. Following gel filtration on Sephacryl S-300, the Tat-associated CTD kinase activity was found in the fractions corresponding to the eluted proteins with a mass of 350 kDa (Fig. 3A, fraction 16–18). Immunoblotting analysis showed that CDK7, cyclin H (Fig. 3A) and MAT1 (not shown) co-eluted with the Tat-associated CTD kinase activity. Fractions 16–18 contain 32, 35, 40, 50 and 60 kDa protein bands (Fig. 3B, protein bands marked by stars). To determine composition of unknown protein bands, fractions 22 to 24 were combined, concentrated on Centricon-10 spin column (Amicon), recovered in SDS-loading buffer and resolved on 12% SDS-polyacrylamide gel. Following staining with colloidal Coumassie blue, only two protein bands of 35 and 50 kDa were visualized and subjected to tryptic digestion and nanoelectrospray MS (described in Experimental procedure section). The 35 kDa protein contained peptides vpflPGDSDlDqltr and YPilENPEilr (lower case letters indicate residues observed with less than full confidence) with sequence identity to CDK7 and cyclin H, respectively. The 50 kDa protein contained a peptide VWDYETGDfER with sequence identity to LIS1.

### HIV-1 Tat interacts with WD domains of LIS1 *in vitro*

**Figure 4:**
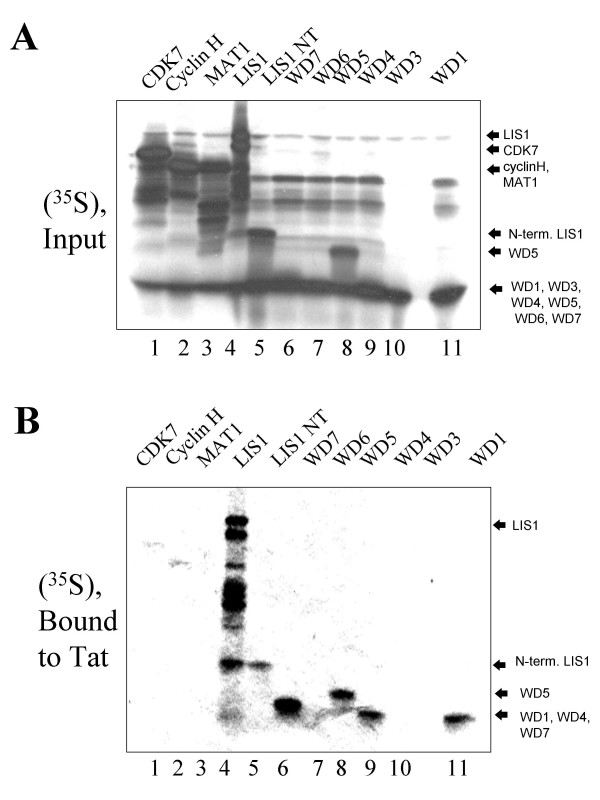
**LIS1 binds to HIV-1 Tat *in vitro*. **Individual protein components of Tat-associated complex were translated in reticulocyte lysate containing [^35^S]methionine as described in the Experimental procedures section. *A*, Input lysates, resolved on 12% SDS-PAGE. *Lane 1*- CDK7; *Lane 2*-Cyclin H; *Lane 3*-MAT1; *Lane 4*-LIS1; *Lane 5*-the N-terminal domain of LIS1 (LIS NT); *Lane 6*- WD7; *Lane 7*-WD6; *Lane 8 *– WD5; *Lane 9 *– WD4; *Lane 10*- WD3; and *Lane 11*- WD1. *B*, programmed reticulocyte lysates from panel A precipitated with GST-Tat 72 immobilized on glutathione-agarose beads, and resolved on 12% SDS-PAGE.

**Figure 5:**
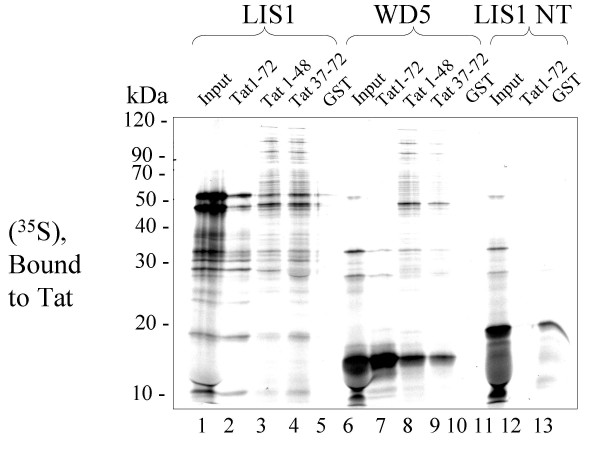
**WD5 domain of LIS1 interact with HIV-Tat. **LIS1, WD5 domain of LIS1 (WD5) and N-terminal portion of LIS1 (LIS1 NT) were translated in reticulocyte lysate containing [^35^S] methionine as described in the Experimental procedures section. Lysates were precipitated with GST-fused Tat 1–72, Tat 1–48, Tat 37–72 or GST alone, immobilized on glutathione-agarose beads, and resolved on 12% SDS-PAGE. *Lanes 1, 6 and 11 *– Input; *Lanes 2, 7 and 12 *– precipitation of LIS1, WD5 or LIS1 NT with Tat 1–72; *Lanes 3 and 8 *– precipitation of LIS1 and WD5 with Tat 1–48; *Lanes 4 and 9 *– precipitation of LIS1 and WD5 with Tat 37–72; *Lanes 5, 10 and 13 *– precipitation of LIS1, WD5 or LIS1 NT with GST alone. The figure is an autoradiogram.

Next we analysed which one of the identified proteins in the elution complex might interact with Tat. We expected that CDK7 might bind to Tat as their interaction was previously reported [35]. We incubated fractions 18 to 24 with GST-fused Tat 1–72, then precipitated GST-Tat with glutathione-agarose beads and analysed associated proteins on SDS-PAGE followed by a silver staining. We found that a 50 kDa protein associated with GST-Tat in fractions 20 and 22 (see Additional file 2, lanes 3 and 4). We then asked whether LIS1, a candidate for a 50 kDa Tat-interacting protein, binds to Tat. We translated LIS1 and also translated as controls CDK7, cyclin H and MAT1, in reticulocyte lysate (Fig. 4A) and performed GST pull down assays using GST-fused Tat 1–72 (Fig. 4B). LIS1 bound to Tat (Fig. 4B, lane 4). In contrast, almost no binding was detected for CDK7, cyclin H or MAT1 (Fig. 4B, lanes 1 to 3). These results contrasted with the previous report in which recombinant Tat interacted with CDK7 immunopurified from reticulocyte lysate [35]. The main difference of our study was that we used programmed lysates rather than purified proteins. Immunoaffinity analysis showed that reticulocyte lysate contains substantial amount of endogenous LIS1 which is comparable to the amount of LIS1 in the LIS1-programmed lysate (see Additional file 3, compare lanes 1–3 to lane 4). Thus the excess of LIS1 might compete for the binding to Tat and prevent CDK7 interaction with Tat. To analyze whether WD domains of LIS1 might associate with Tat, we expressed each of WD domain, except domain 2 as well as the N-terminal part of LIS1, which contains a coiled-coiled motif and which is devoid of WD domains. The WD domain 1, 4, 5 or 7 bound to Tat (Fig. 4B, lanes 6 to 11). Also the N-terminal portion of LIS1 bound weakly to Tat (Fig. 4B, lane 5). To analyze specificity of the binding and to determine a domain of Tat that binds LIS1, several Tat mutants were utilized including Tat 1–72, Tat 1–48, and Tat 37–72 and also GST as a control (Fig. 5). Full length LIS1 bound with equal efficiency to a full length Tat, Tat 1–48 or Tat 37–72 but not to GST alone (Fig. 5, lanes 2 to 5). In contrast, isolated WD5 domain of LIS1 bound most efficiently to the full length Tat 1–72 and less efficiently to Tat 1–48 or to Tat 37–72 (Fig. 5, lanes 6 to 9). The isolated N-terminal domain of LIS1 bound strongly to GST (Fig. 5, lane13), and thus its weak binding to GST-Tat (Fig. 5, lane 12) is likely to be mediated by the binding to the GST moiety.

### Tat co-immunoprecipitates with LIS1 from HeLa cellular extracts

**Figure 6:**
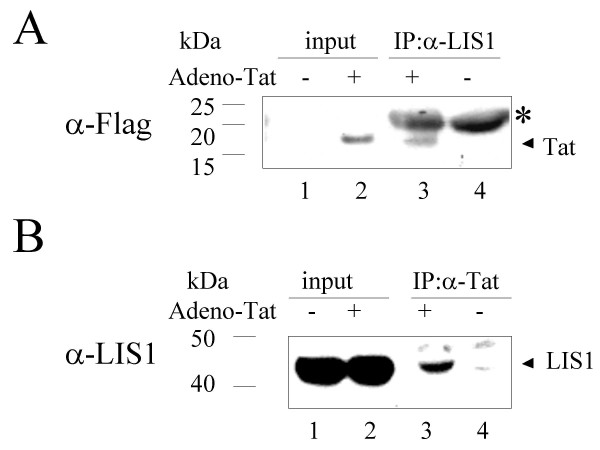
**Co-immunoprecipitation of HIV-1 Tat with LIS1 from HeLa cells. **HeLa whole cell extracts, with and without Flag-Tat, were prepared from uninfected and Adeno-Tat infected cells as described in the *Experimental procedures *section. *A*, LIS1 was immunoprecipitated with monoclonal anti-LIS1 antibodies, resolved by 10% Tris-Tricine gel and immunoblotted with anti-Flag antibodies to detect Flag-Tat. *B*, Flag-Tat was immunoprecipitated with polyclonal anti-Flag antibodies, resolved by 12% Tris-Tricine gel and probed with monoclonal anti-LIS1 antibodies to detect LIS1.

To analyze interaction of Tat with LIS1 in cultured cells, co-immunoprecipitation analysis was performed. Tat was expressed in HeLa cells infected with adenovirus vector expressing Flag-tagged Tat [36]. Tat expression in the extract was verified by immunoblotting analysis with anti-Flag antibodies (Fig. 6A, compare lane 2 to lane 1) and also with anti-Tat antibodies (not shown). LIS1 was expressed equally in control cells without Tat and in the cells expressing Flag-Tat (Fig. 6B, lanes 1 and 2). Tat co-precipitated with LIS1 when LIS1 was immunoprecipitated with LIS1-specific monoclonal antibodies, resolved by 12% Tris-Tricine PAGE and immunoblotted with anti-Flag antibodies (Fig. 6A, lane 3). No Tat was detected in the control immunoprecipitation (Fig. 6A, lane 4). Similar, LIS1 co-precipitated with Tat when Flag-Tat was immunoprecipitated with anti-Tat polyclonal antibodies, resolved by 10% Tris-Tricine PAGE and immunoblotted with anti-LIS1 antibodies (Fig. 6B, lane 3). No LIS1 was detected in the control immunoprecipitation (Fig. 6B, lane 4). These results indicate that Tat associates with LIS1 in cultured cells.

### Tat binds to LIS1 in yeast two-hybrid system

**Figure 7:**
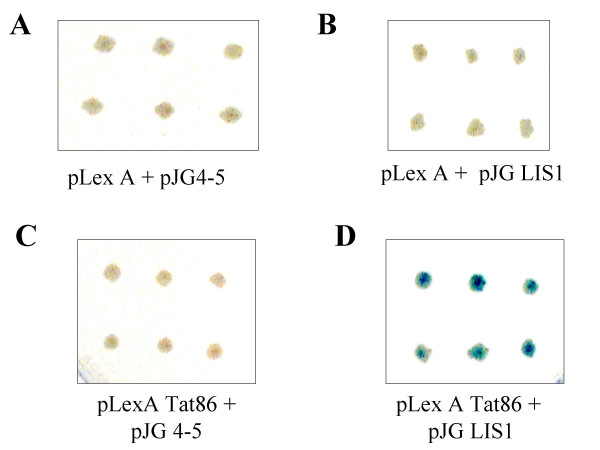
**LIS1 interacts with Tat in yeast two-hybrid assay. **EGY48 yeast cells were transformed, as described in *Experimental procedures*, with pSH18–34 reporter and combinations of pLexA and pJG4–5 empty vectors (*panel A*); pLexA and pJG LIS1 (*panel B*); pLexA Tat and pJG 4–5 (*panel C*); pLexA Tat 86 and pJG LIS1 (*panel D*). Six independent colonies from each transformation were cultured on plates containing Galactose/Raffinose to induce Tat and LIS1 synthesis and X-Gal substrate to detect β-galactosidase.

To analyze whether Tat interacts with LIS1 directly and not through another protein, we utilized LexA-based yeast two hybrid system (Clontech, see details in *Experimental procedures*). EGY48 yeast cells pretransformed with pSH18–34 reporter plasmid (-Ura selection) were further transformed with different combinations of pJG-LIS1 or pJG4–5 empty vector (-Trp selection) and pLexA Tat or pLexA empty vector (-His selection). Colonies grown on-His/-Trp/-Ura media with glucose were plated on Galactose/Raffinose His /-Trp/-Ura plates, to induce LIS1 and Tat production. The plates also contained 5-Bromo-4-Chloro-3-Indolyl-β-D-galactopyranoside (X-Gal) substrate for β-galactosidase. Tat interacted with LIS1 as it was detected by development of blue color upon conversion of X-gal (Fig. 7D). In contrast Tat did not interact with the acid activation domain alone (Fig. 7C). Also no interaction was detected for LexA DNA binding domain and acid activation domain (Fig. 7A) or LexA DNA binding domain and LIS1 (Fig. 7B).

Taken together, these results indicate that LIS1 directly and specifically binds to Tat *in vivo*.

## Discussion

In this study, we show that HIV-1 Tat protein associates with LIS1 protein. LIS1, a microtubule binding protein [21] contains WD repeats [24] that are likely to participate in protein-protein interactions [24]. LIS1 regulates microtubule dynamics by interacting with dynein motor, NudC and Dynactin [27,28] and also with Nudel [29]. A yeast homologue of LIS1, NudF associates with NudC to regulate dynein and microtubule dynamics [37,38]. Thus, interaction of Tat with LIS1, a scaffold that assembles dynein motor, may affect microtubule dynamics.

We purified several candidate proteins that might interact with Tat, and found CDK7, cyclin H, MAT1 and LIS1. We expected that CDK7 might bind to Tat as previously it was shown to interact directly with Tat [35]. In contrast, analysis of the binding of individually translated proteins showed that LIS1 and not CDK7 bound to Tat. We hypothesized that WD domain(s) of LIS1 might bind Tat, as these domains form a planar surface. Correspondingly, domains WD1, WD4, WD5 and WD7 were found to bind Tat but not the N-terminal part of LIS1 that contains coil-coil region, and which is devoid of WD domains. We analyzed whether a particular domain of Tat binds LIS1 or WD5 domain of LIS1. Full length Tat 1–72 was most efficient in binding of either LIS1 or WD5 domain of LIS1. It would be interesting to determine whether CDK7 also binds to LIS1, and whether LIS1 promotes activation of the kinase activity of CDK7 by Tat. Although LIS1 is a cytoplasmic protein, it may be required for initial assembly of a protein complex containing CDK7. Our results contrasted with the previous report in which Tat binds to purified CDK7 [35]. We hypothesize that under our experimental conditions, excess of endogenous LIS1 present in the reticulocyte lysate might compete with interaction of Tat with CDK7. Interestingly, Gaynor an colleagues only detect specific interaction of Tat with TFIIH but not with of CDK7 or CAK alone [39]. Therefore, it is possible that in a complex protein mixture Tat interacts with CDK7 indirectly through another protein such as LIS1.

To explore interaction of Tat and LIS1 in cultured cells, Flag-tagged Tat was expressed in HeLa cells and then immunoprecipitated with anti-Flag-antibodies. LIS1 was found to co-immunoprecipitate with Tat. Correspondingly, when LIS1 was immunoprecipitated with anti-LIS1 monoclonal antibodies, Flag-Tat was found in the immunoprecipitates. These results suggest that Tat associates with LIS1 in cultured cells. To confirm that LIS1 and Tat interact *in vivo*, we used yeast two-hybrid system, in which Tat was expressed as a bait and LIS1 as a prey. Again, we found that LIS1 and Tat interacted in this system. Taken together, our *in vitro *and *in vivo *results demonstrate that HIV-1 Tat binds to LIS1 and that this binding is likely to occur through one of the WD domains of LIS1.

Tat contains several functionally important regions, including the N-terminal region I (residues 1–21); cystein-rich region II (residues 22–37); core region III (residues 38–48); basic region IV (residues 49–59); glutamine-rich region V (residues 60–72); and C-terminal region VI [20,40]. Zhou and his colleagues showed that Tat interacts with microtubules through parts of region II (residues 35–37) and region III (residue 38) [18]. More recently, Loret and his colleagues showed that the glutamine-rich region of Tat may also interact with microtubules and promote apoptosis in T cells [20]. In a following study which will appear in the same issue of Retrovirology, Loret and his colleagues show that Tat residues 38–72 are sufficient to enhance microtubule polymerization and that the extent of the enhancement correlates with the severity of Tat-induced apoptosis[41]. Taken together these studies indicate that residues 35–38 of regions II and III and glutamine-rich region of Tat may interact with microtubules. These results correlate well with our finding that full length Tat binds LIS1 better than the isolate domains of Tat. Whether LIS1, a cellular structural protein and also an enzymatic subunit of PAF-AH, plays a role in Tat-induced apoptosis remained to be determined. As Tat-associated proteins include CDK7, Cyclin H, MAT1 and LIS1, it is possible that interaction of Tat with LIS1 might promote binding of CDK7 and ultimately affect viral gene expression through a direct activation of CDK7 or indirectly through activation of a down stream kinase, CDK2, by CDK7. As Tat is shuttling between nucleus and cytoplasm, its interaction with LIS1 and CDK7-containing protein complex might allow a temporary activation/modulation of the CDK7 activity. It is remained to be determined whether such interaction has an effect on Tat-induced transcription of HIV-1 genes. LIS1 may also function as an adaptor that brings HIV-1 Tat to microtubules that may release microtubules-associated BIM-1 protein and induce apoptosis [18]. A more detailed future study will address the questions of the regulation of HIV-1 transcription and Tat-mediated apoptosis by LIS1.

## Methods

### Materials

Jurkat T-cells were purchased from National Cell Culture Center (CELLEX BIOSCIENCES, MN). DEAE-Sepharose (FF), SP-Sepharose (FF), Hi Trap heparin columns, [γ-^32^P] ATP (6000 Ci/mmol) and (^35^S)-labeled Methionine were purchased from Amersham Pharmacia Biotech (Piscataway, NJ). Econo-Pac CHT-II Cartridge (ceramic hydroxyapatite) was from Bio-Rad (Hercules, CA). Glutathion-agarose was from Sigma (Atlanta, GA). GST-CTD was expressed in *Escherichia coli *and purified as we described [32]. The Tat expression plasmids GST-Tat (1–72), GST-Tat (1–48), GST-Tat (37–72) were obtained from AIDS Research and Reference Reagents Program (NIH), expressed in *Escherichia coli *and purified on Glutathione-agarose beads as described [31]. CDK7, cyclin H and MAT1 expression vectors were kindly provided by Dr. Marcel Doreé (CNRS, Montpellier, France). Coupled transcription/translation system based on rabbit reticulocyte lysate was purchased from Ambion (Austin, TX). Protein (G) and protein (A) agarose were purchased from Sigma (Atlanta, GA).

### Antibodies

Anti-Tat rabbit polyclonal (HIV-1 BH10 Tat antiserum) and monoclonal (NT3 2D1.1) antibodies were received from AIDS Research and Reference Reagents Program (NIH). Anti-Flag antibodies were purchased from Sigma (Atlanta, GA). Polyclonal antibodies to CDK7, and PSTAIRE were purchased from Santa Cruz Biochemical (Santa Cruz, CA). Polyclonal antibody to CDK9 (PITALRE) were purchased from Biodesign Company (Saco, ME). Monoclonal antibodies for LIS1 were as described [21].

### Tat-associated CTD kinase assay

Tat-associated kinase activity was assayed as described previously [32]. Briefly, portions of eluted fractions (about 1/1000 of the total amount) from each chromatography column were incubated with 10 μg of GST-Tat (1–72) immobilized on glutathione-agarose beads for 1 hour at 4°C. The beads were washed with the buffer B containing 20 mM HEPES (pH 7.9), 250 mM NaCl, 1% NP-40, 5 mM EDTA, 0.5 mM DTT, 0.5 mM PMSF and 10 μg/ml aprotinin, followed by washing with the kinase buffer (50 mM HEPES (pH 7.9), 10 mM MgCl_2_, 6 mM EGTA and 2.5 mM dithiothreitol). Tat-associated CTD kinase activity was assayed by incubating the kinase-bound beads with 100 ng GST-CTD in kinase buffer containing 50 μM ATP and 10 μCi of (^32^p)ATP for 10 min at room temperature. Phosphorylated GST-CTD was resolved on 10% SDS-PAGE and subjected to autoradiography and quantification with PhosphorImager Storm 860 (Molecular Dynamics).

### Purification of Tat-associated CTD kinase

Purification of Tat-associated CTD kinase from Jurkat T-cells was carried as previously described [32]. Briefly, 100 liters of Jurkat T cell culture at concentration of 5 × 10^5 ^cells/ml were centrifuged, washed and Dounce-homogenized in Buffer A (50 mM HEPES [pH 7.9], 5 mM EDTA, 0.5 mM DTT, 0.5 mM PMSF, 10 μg/ml aprotinin and 10% glycerol) supplemented with 0.1% NP-40. The whole cell extract was prepared and fractionated by ammonium sulfate precipitation. Ammonium sulfate was added to 10% saturation to extract nuclei. After centrifugation, the supernatant, containing approximately 10 g of protein, was further fractionated with ammonium sulfate added to 20%, 40% and 80% saturation. The 40% ammonium sulfate fraction (about 3.5 g of protein) was found to contain the major part of Tat-associated CTD kinase activity. This fraction was diluted with Buffer A until the conductivity was equivalent to 50 mM KCl and then loaded on a DEAE-Sepharose column (about 500 mg of protein per 50 ml column). The column was eluted with a linear gradient of KCl (0.1 to 1 M) in Buffer A. Fractions were assayed for Tat-associated CTD kinase activity as described above. A peak of Tat-associated CTD kinase activity was collected, diluted with Buffer A until conductivity was equivalent to 50 mM KCl and loaded on a 10 ml SP-Sepharose column which was eluted with linear gradient of KCl (0.1 to 1 M) in Buffer A. A flow-through fraction containing Tat-associated CTD kinase activity was further fractionated on Hi Trap heparin columns (1 ml, three in series). Fractions were collected and analyzed for the Tat-associated CTD-kinase activity as described above, as well as by immunoblotting. Fractions containing Tat-associated CTD kinase activity TTK were resolved on 12% SDS-PAGE (20 × 20 cm) stained with colloidal Coumassie Blue and subjected to protein microsequencing.

### NanoLC ion trap mass spectrometry and peptide sequencing

The procedure for peptide sequencing was performed as described previously. Protein bands visible after colloidal Coomassie blue staining and corresponding to the peak of CTD hyperphosphorylating activity after the heparin-agarose column were subjected to in-gel reduction, carboxyamidomethylation and tryptic digestion (Promega, Madison, WI). Multiple peptide sequences were determined in a single run by microcapillary reverse-phase chromatography directly coupled to a Finnigan LCQ quadrupole ion trap mass spectrometer equipped with a custom nanoelectrospray source. The column was packed in-house with 5 cm of C18 support into a New Objective one-piece 75 um I.D. column terminating in a 15 μm tip. Flow rate was 190 nanoliters/min. The ion trap was programmed to acquire successive sets of three scan modes consisting of full scan MS over alternating ranges of 395–800 m/z or 800–1300 m/z, followed by two data dependent scans on the most abundant ion in those full scans. These data dependent scans allowed the automatic acquisition of a high resolution (zoom) scan to determine charge state and exact mass, and MS/MS spectra for peptide sequence information. MS/MS spectra were acquired with a relative collision energy of 30%, an isolation width of 2.5 Dalton and recurring ions dynamically excluded. Interpretation of the resulting MS/MS spectra of the peptides was facilitated by programs developed in the Harvard Microchemistry Facility and by database correlation with the algorithm SyQuest [42].

### In vitro proteins synthesis

Proteins were transcribed/translated as described previously [32]. Briefly, the CDK7, cyclin H and MAT1, LIS1 and different domains of LIS1 were transcribed/translated in a coupled rabbit reticulocyte system according to manufacturer recommendations (Ambion, Austin, TX). Proteins were resolved on 12% SDS-PAGE. The gel was treated with Amplify solution (Amersham Pharmacia Biotech, Piscataway, NJ), dried and exposed to X-ray film with intensifying screen at -70°C.

### Co-immunoprecipitation and Western blot

HeLa cells were infected with adenovirus vector expressing Flag-tagged Tat protein as we previously described [36]. HeLa whole cell extracts were prepared as described previously [43]. Cell extracts were also prepared from non-infected HeLa cells and used as a control. About 100 μg of whole cell extract was supplemented with 5 μg of anti-Flag or anti LIS1 antibodies. Then protein G-agarose beads preblocked with 5% BSA and suspended in TNN buffer (50 mM Tris-HCl (pH 7.5), 0.5% NP-40, 150 mM NaCl) buffer were added and the reaction was incubated in TNN buffer at 4°C for 2 h with rocking. The beads were precipitated and washed once with TNN buffer and once with the kinase buffer (50 mM HEPES-KOH (pH-7.9), 10 mM MgCl_2, _6 mM EGTA, 2.5 mM DTT). The pellet was then resuspended in a 30 μl of 1X SDS loading buffer (4% SDS, 10% glycerol, 5% 2-mecarpthaethanol, 0.002% bromophenol blue) and heated at 90°C for 3 minutes. The proteins were resolved on SDS Tris-Tricine PAGE, 10%, to detect LIS1, or 12%, to detect Tat, and immunoblotted with anti-LIS1 or anti-Flag antibodies.

### Yeast two-hybrid system

The parent yeast cells EGY48 (LexA 2H) genotype (*MATα*, *ura3*, *his3*, *tryp1*, *LexA*_*op *(*x*6) _-*LEU2*), auxotrophic for tryptophan (Trp), uracil (Ura), histidine (His), with LEU2 as a reporter gene. Yeast were transformed by electroporation as follow. One colony of the yeast cells was resuspended into 10 ml of appropriate selective media and grown at 30°C overnight. Cells were collected at 3000 rpm for 10 min, washed twice with HEPES/Sorbitol (20 mM HEPES pH 7.9, 1 M Sorbitol), resuspended in 200 μl of HEPES/Sorbitol and supplemented with 1 μg of a plasmid DNA. The mixture was pulsed with 2500 V in 0.4 cm cuvette, then 1 ml of appropriate selective media was added and cells were shaken at 30°C for 2 hours. The cells were collected by centrifugation, resuspended into 250 μl of HEPES/Sorbitol and plated on appropriate selective plates. EGY48 cells were transformed with pSH18–34 vector containing *Lac Z *reporter under the control of LexA_op(x8) _and also *URA3 *and *amp*^*r *^genes as selection markers. The transformed yeast cells (EGY48-lacZ) were selected on Uracyl deficient media. HIV-1 Tat first exon was subcloned into pLexA in frame with the LexA_(1–202)_, the DNA binding domain to create the bait vector (pLexA-Tat). LIS1 was subcloned into pJG 4–5 (*amp*^*r*^) in frame with the acid activation domain to create pJG-LIS1 carrying hemagglutinin (HA) tag (Trp selectable marker). The EGY48-lacZ yeast cells were transformed with pLexA-Tat vector, and selected for growth on uracyl and histidine deficient media. The Tat expressing yeast cell growing on Uracyl, Histidine deficient plates were then transformed with pJG-LIS1. To detect interaction between Tat and LIS1 interaction, yeast cells were plated on galactose/raffinose-containing plates to allow expression of Tat and LIS1, and production of β-galactosidase was visualized with 5-bromo-4-chloro-3-indolyl-β-D-galactoside (X-gal) substrate.

## Competing interests

The author(s) declare that they have no competing interests.

## Authors' contributions

NE carried out studies of LIS1 and Tat interaction *in vitro *and *in vivo *and participated in the writing and assembling of the manuscript. TA carried out yeast two-hybrid assays. YV provided technical help. WSL performed protein sequencing. WT participated in the design and discussion of the study. TS and OR created vectors for expression of LIS1 and participated in the design of the study. SN purified LIS1 containing protein complex for protein sequencing, performed general control and coordination of the study. All authors read and approved the manuscript.

## Supplementary Material

Additional File 1 **Analysis of protein composition of DEAE-Sepharose purified fraction of Tat-associated CTD kinase. **Fractions from the DEAE-Sepharose column fractionation shown in Fig. 1B were analyzed for Tat-associated CTD kinase activity and also by Western blotting with antibodies against CDK7, CDK9, p62 subunit of TFIIH and PSTAIRE. Click here for file

Additional File 2 **HIV-Tat interacts with a 50 kDa protein from purified Tat-associated CTD kinase. **GST-fused Tat 1–72, immobilized on glutathione-agarose beads, was incubated without (lane 1), or with fraction 18 (lane 2), fraction 20 (lane 3), fraction 22 (lane 4), or fraction 24 (lane 5) from the heparin-agarose, shown in Fig. 2B. Precipitated Click here for file

Additional File 3 **Endogenous LIS1 is present in reticulocyte lysates. **Individual protein components of Tat-associated complex were translated in reticulocyte lysate. The lysates were resolved on 12% SDS-Tris-Tricine gel and immunoblotted with anti-LIS1 monoclonal antibodies. *Lane 1*- CDK7; *Lane 2*-Cyclin H; *Lane 3*-MAT1; and *Lane 4*-LIS1-programmed lysate. Click here for file
