# Supplementary figures and images for: HIV-1 Tat interacts with LIS1 protein
Source: Retrovirology. 2005 Feb 7;2:6. doi: 10.1186/1742-4690-2-6 (PMC549217; doi:10.1186/1742-4690-2-6)

# DEAE-Sepharose Fractions

Kinase Assay

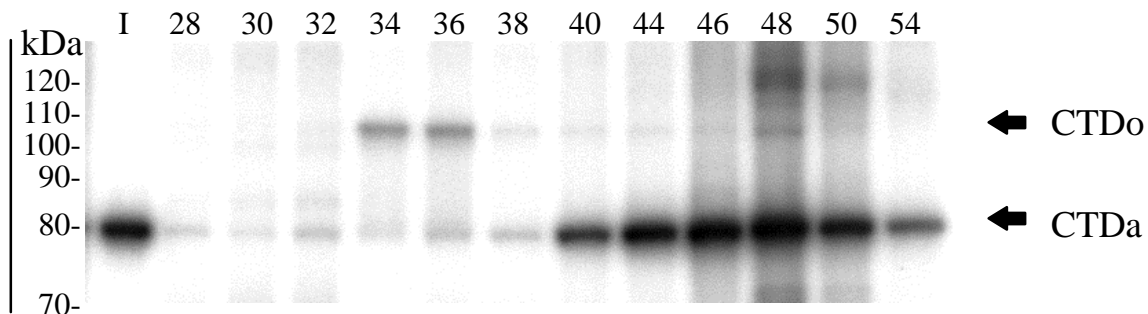

$\alpha$ -CDK7

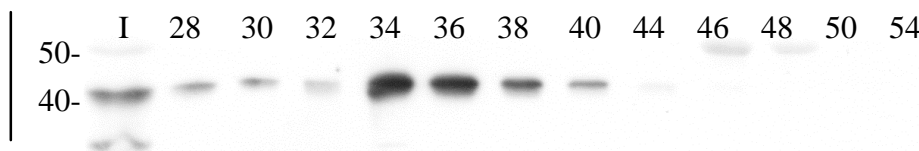

$\alpha$ -CDK9

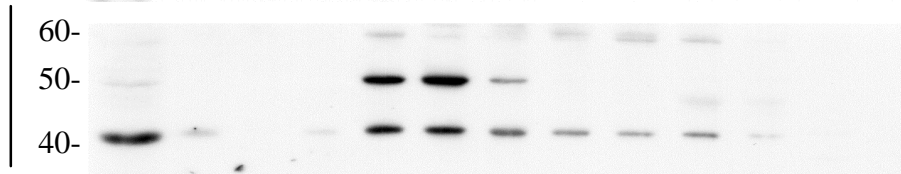

$\alpha$ -TFIIH,  
p62

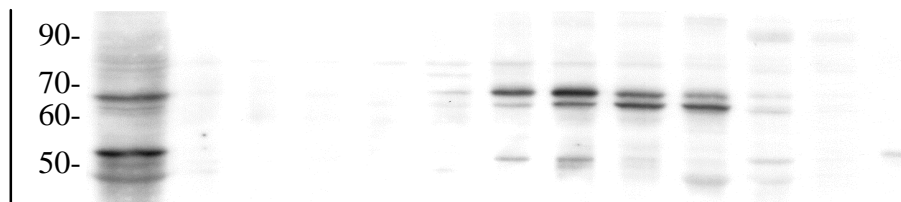

$\alpha$ -PSTAIR

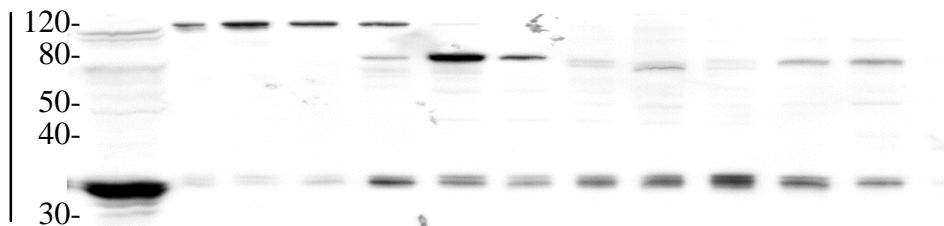

TTK

Supplement: Additional File 1 — Analysis of protein composition of DEAE-Sepharose purified fraction of Tat-associated CTD kinase. Fractions from the DEAE-Sepharose column fractionation shown in Fig. 1B were analyzed for Tat-associated CTD kinase activity and also by Western blotting with antibodies against CDK7, CDK9, p62 subunit of TFIIH and PSTAIRE. [file 1742-4690-2-6-S1.pdf]

Heparin-agarose fractions  
Added to GST-Tat72

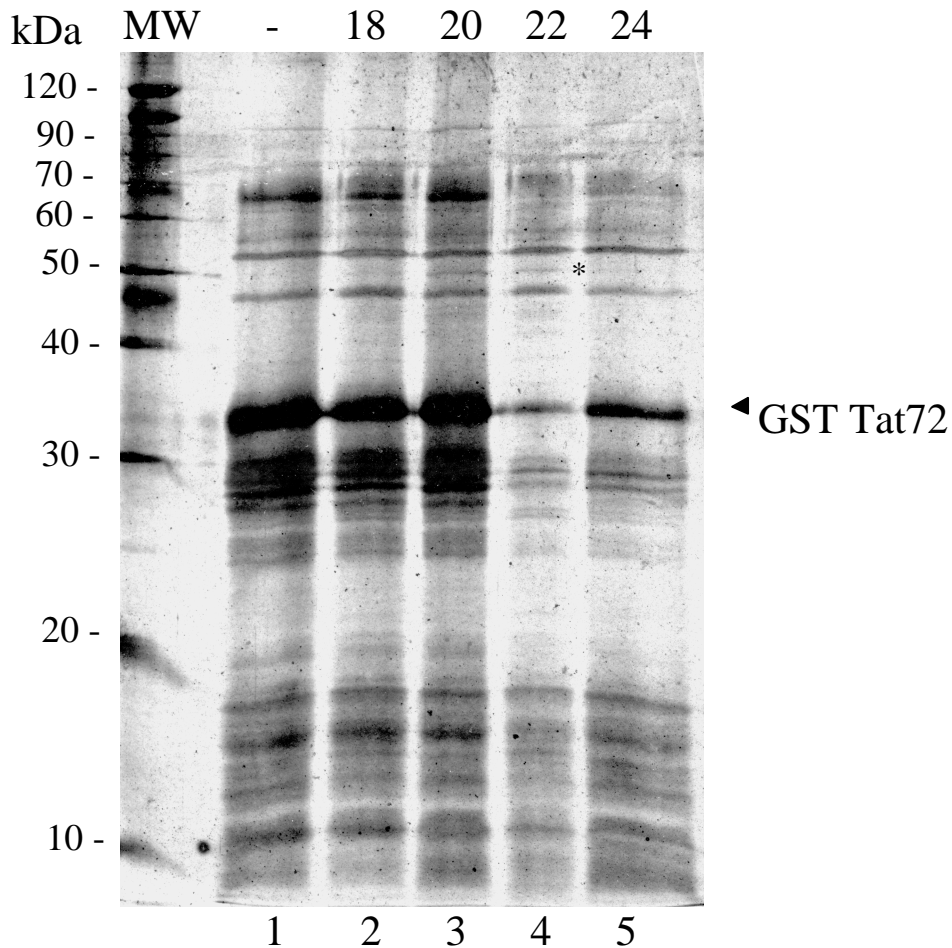

Supplement: Additional File 2 — HIV-Tat interacts with a 50 kDa protein from purified Tat-associated CTD kinase. GST-fused Tat 1–72, immobilized on glutathione-agarose beads, was incubated without (lane 1), or with fraction 18 (lane 2), fraction 20 (lane 3), fraction 22 (lane 4), or fraction 24 (lane 5) from the heparin-agarose, shown in Fig. 2B. Precipitated [file 1742-4690-2-6-S2.pdf]

CDK7

+

-

-

-

Cyclin H

-

+

-

-

MAT1

-

-

+

-

LIS1

-

-

-

+

$\alpha$ -LIS1

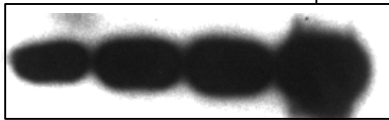

◀ LIS1

1

2

3

4

Supplement: Additional File 3 — Endogenous LIS1 is present in reticulocyte lysates. Individual protein components of Tat-associated complex were translated in reticulocyte lysate. The lysates were resolved on 12% SDS-Tris-Tricine gel and immunoblotted with anti-LIS1 monoclonal antibodies. Lane 1- CDK7; Lane 2-Cyclin H; Lane 3-MAT1; and Lane 4-LIS1-programmed lysate. [file 1742-4690-2-6-S3.pdf]
